# Surgery, radiotherapy and endocrine therapy for oligometastatic prostate cancer efficacy: a systematic review and network meta-analysis

**DOI:** 10.7717/peerj.19819

**Published:** 2025-08-29

**Authors:** Wenwei Ying, Zhenshan Ding, Yuhui He, Jianfeng Wang, Xing Chen, Xuesong Li, Yanqing Gong

**Affiliations:** 1 Department of Urology, Peking University First Hospital, Beijing, China; 2 Institution of Urology, Peking University, Beijing, China; 3 Beijing Key Laboratory of Urogenital Diseases (Male) Molecular Diagnosis and Treatment Center, Beijing, China; 4 National Urological Cancer Center, Beijing, China; 5 Department of Urology, China-Japan Friendship Hospital, Beijing, China

**Keywords:** Oligometastatic prostate cancer, Surgery, Radiotherapy, Drug endocrine therapy, Network meta-analysis, Efficacy, Adverse events

## Abstract

In recent years, the treatment approach for metastatic prostate cancer has evolved, with early combination therapies increasingly being favored over androgen-deprivation therapy (ADT) alone. Despite the availability of various treatments, their relative effectiveness and safety trade-offs remain uncertain. Randomized controlled trials have explored a range of treatments for oligometastatic prostate cancer, but clear conclusions regarding their prognostic benefits and patient-centered outcomes have not been established. This network meta-analysis (NMA) aims to quantify the benefits of different treatments by analyzing data from a systematic search of Medline, EMBASE, and Cochrane databases, covering trials up to October 1, 2024. The primary outcomes evaluated in this study include overall survival (OS), progression-free survival (PFS), treatment-related adverse events (TRAEs), and quality of life (QoL). This study is registered with PROSPERO (CRD42022370203). We analyzed individual patient data from 13 eligible trials, involving a total of 2,524 patients. Our analysis revealed that ADT+ radiation therapy (RT) (hazard ratio (HR) = 0.39, 95% confidence interval (CI) [0.27–0.56]) and ADT+stereotactic body radiotherapy (SBRT) (HR = 0.35, 95% CI [0.21–0.58]) significantly improved progression-free survival (PFS) compared to ADT alone, while no treatment showed a significant overall survival (OS) benefit. Safety analysis revealed ADT monotherapy had the lowest risk of grade ≥3 adverse events (TRAEs), whereas ADT+abiraterone increased toxicity (OR = 1.54). Limited quality of life (QoL) data suggested ADT+RT may offer slight improvement (surface under the cumulative ranking curve (SUCRA) 74.3%). Most trials exhibited low bias risk, though heterogeneity and small sample sizes for some comparisons warrant cautious interpretation. These findings support ADT+RT/SBRT for PFS benefit but highlight the need for further research to optimize survival outcomes and treatment tolerability.

## Introduction

Oligometastatic prostate cancer (omPCa) has garnered increasing attention in recent years, with a growing body of prospective studies exploring its management. Androgen deprivation therapy (ADT) and other endocrine therapies remain the cornerstone of treatment for metastatic prostate cancer, representing a pivotal advancement in the care of advanced disease by substantially improving patient survival (Cornford et al., 2017). In contrast, surgical interventions are seldom recommended in this setting.

A recent review has challenged conventional treatment paradigms, reporting a 5-year overall survival rate of 55% for surgery, compared to 25.3% for radiotherapy and 41.3% for endocrine ADT in oligometastatic prostate cancer (Tosoian et al., 2017). These findings suggest a potential survival benefit with surgical intervention, yet the evidence remains insufficiently explored. Consequently, the optimal therapeutic strategy—whether surgery, radiotherapy, or endocrine therapy—for improving prognosis in oligometastatic prostate cancer remains unresolved.

To address this uncertainty, we conducted a systematic review and network meta-analysis (NMA) to evaluate the comparative efficacy of surgical, radiotherapeutic, and pharmacological endocrine therapies in oligometastatic prostate cancer. Our aim was to synthesize existing evidence and identify the most effective treatment approach for this patient population.

## Methods

The methodology for this systematic review and network meta-analysis (NMA) was adapted from established protocols described in prior research (Page et al., 2021), with adherence to the Preferred Reporting Items for Systematic Review and Meta-Analysis extension for Network Meta-Analyses (PRISMA-NMA) guidelines. The study protocol followed standard registration procedures through PROSPERO (CRD42022370203).

This framework structured the Medline, EMBASE, and Cochrane database searches into three conceptual tiers: (1) intervention terms (“Radical prostatectomy,” “Radiotherapy,” “Androgen receptor axis-targeted therapy”), (2) condition terms (“Prostate Neoplasms” AND “Oligometastatic”), and (3) study design filters (“Randomized Controlled Trial”). The full search syntax with Medical Subject Headings (MeSH) terms and keyword permutations has been archived on INPLASY Protocol to ensure reproducibility (Ying et al., 2022). Inclusion criteria were restricted to randomized two-arm trials evaluating active interventions for oligometastatic prostate cancer.

We included patients with oligometastatic prostate cancer in randomized controlled trials and combined androgen-deprivation therapy (ADT) with one or more of the previously listed treatments. In the 13 articles eventually included, the diagnosis of oligometastatic prostate cancer was based on conventional imaging techniques (such as scintigraphic bone scans, thoracic computed tomography (CT), abdominal CT, and pelvic magnetic resonance imaging (MRI) or CT), as well as histological and/or cytological confirmation.

Initially, two independent authors (Wenwei Ying and Zhenshan Ding) performed the search strategy and screened the search results for relevance based on the topics and abstracts. Any disagreements between them were resolved by consulting a third author (Yanqing Gong). The full texts of the 58 articles identified as potentially relevant were then reviewed by the same two independent authors (Wenwei Ying and Zhenshan Ding) to determine if they met the predefined inclusion and exclusion criteria. If multiple reports of the same trial were found, only the most recent publication was included in the analysis. Data extraction was also conducted by the two independent authors (Wenwei Ying and Zhenshan Ding).

The primary outcome was overall survival (OS), measured as the time from randomization to death from any cause. We also evaluated secondary endpoints, including progression-free survival (PFS), defined as the time from enrollment to prostate-specific antigen (PSA) progression, quality of life (QoL), and treatment-related adverse events (TRAEs).

### Statistical analysis

All statistical analyses were conducted using Stata (version 15.0) and R (R Foundation for Statistical Computing, Vienna, Austria, version 4.1.0). For categorical variables, we calculated the pooled odds ratio (OR) with a 95% confidence interval (95% CI). For continuous variables, we computed the pooled standardized mean difference (SMD) with 95% CI. A virtual study comparing ADT and MDT was added to connect isolated subnetworks, with parameters set to neutral effect (SMD = 0) and wide confidence intervals to minimize its impact. The network diagram was used to visualize direct comparisons among different treatments, with node sizes reflecting the sample sizes of each intervention and the thickness of the connecting lines representing the number of studies comparing the interventions directly. We evaluated global inconsistency using the design-by-treatment interaction model, and local inconsistency was assessed using the node-splitting method to ensure that the direct and indirect estimates were consistent. A *p*-value greater than 0.05 indicated no significant differences between direct and indirect estimates, allowing the use of the consistency model; otherwise, the inconsistency model was utilized. Network heterogeneity was assessed across all treatment comparisons using the I^2^ statistic, while loop-specific heterogeneity was evaluated using the *τ*^2^ statistic. To rank the interventions in terms of efficacy and safety, we calculated the surface under the cumulative ranking curve (SUCRA) values for the primary and secondary outcomes. Additionally, we created league tables that summarized both direct and indirect comparisons for each outcome measure. Risk of bias was assessed using the Cochrane framework (Higgins et al., 2011). The NMA was conducted using the R package “netmeta” (version 2.9-0, R Foundation), “gemtc” (version 1.0-1, R Foundation) and “rjags” (version 4.3.0, R Foundation).

## Results

**Figure 1:**
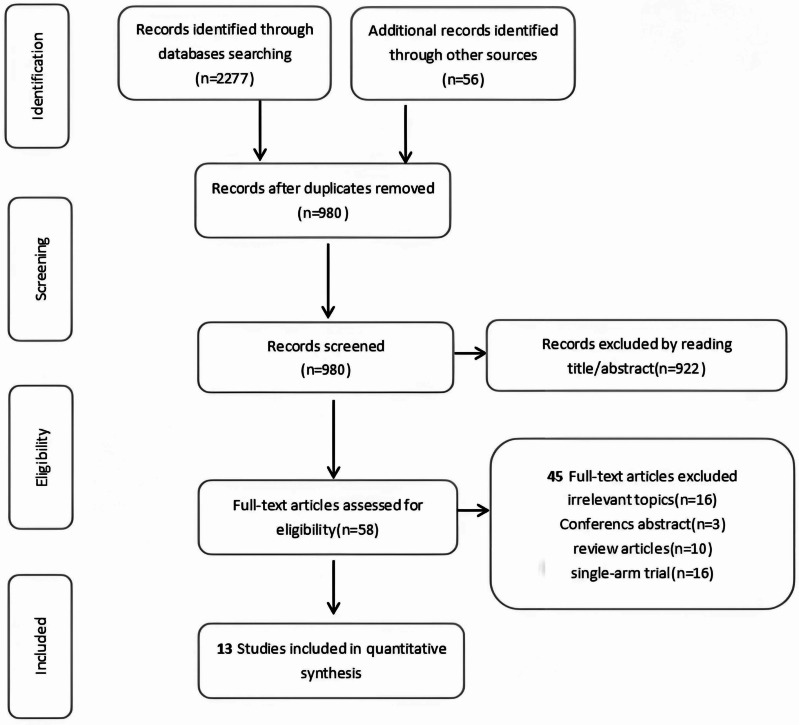
PRISMA flowchart. Preferred reporting items for systematic reviews and meta-analyses (PRISMA), the flowchart of the study process.

After a rigorous screening process involving abstract review and full-text assessment, 13 eligible studies comprising 2,524 patients were ultimately included in this systematic review and network meta-analysis (Fig. 1). Table 1 presents the detailed clinical characteristics of the included studies. The investigated interventions encompassed a range of treatment modalities: observation, androgen-deprivation therapy (ADT), external beam radiation therapy (EBRT), metastasis-directed therapy (MDT), radiation therapy (RT), stereotactic ablative radiotherapy (SABR), as well as combination therapies such as ADT+RT, ADT+SABR, ADT+abiraterone, ADT+enzalutamide (ENZA), ADT+EBRT, and ADT+abiraterone+RT.

**Table 1:** Characteristics of the included randomized control trials.

First author	Year	Country	Trial type	Registration number trial name	Disease stage	Control arm treatment	Patients in control arm (n)	Age	PSA median (range)	Gleason grade group	Experimental arm treatment	Patients in the experimental arm (n)	Age	PSA level, ng/mL	Gleason grade	Median follow-up
Chad Tang^1^	2023	U.S.A	Prospective, Phase II Trial	EXTEND	NA	Hormone therapy only	44	67 (63–72)	≤0.2 (61) >0.2 to <2.0 (32) ≥2.0 (7)	NA	Radiation therapy + hormone therapy	43	67 (63–72)	≤0.2 (53) >0.2 to <2.0 (35) ≥2.0 (12)	NA	22.0 mo (range, 11.6– 39.2 mo)
Bo Dai^2^	2022	China	Prospective, Phase II Trial	NCT02742675	T2c 34 T3 133 T4 33	ADT	100	69 (64–73)	102 (49–254)	≤7 (12) 8–10 (85)	ADT + RLT	100	67 (62–71)	90 (35–236)	≤7 (14) 8–10 (86)	48(IQR, 43-50)mo
Prasanna Sooriakumaran^3^	2022	UK	Prospective	TRoMbone	T2c 5 (10) T3 45 (90)	SOC	25	66.0 (60.2–71.2)	16.5 (8.2–37.5)	=7 7(28) =8 6 (24) 9–10 12 (48)	SOC + radical prostatectomy	25	65.4 (62.5–69.3)	14.0 (2.7–40.0)	=7 7(28) =8 7 (28) 9–10 11 (44)	NA
Claire Petit^4^	2023	Canada	Prospective, Phase II Trial	NCT03525288	NA	Intensified RT+SOC	12	NA	8.0 (1.9–105.0)	=7 6(50) =8 3(25) ≥9 3 (25)	PSMA PET/ CT +SOC	11	NA	6.7 (0.4–33.0)	=6 1(9.1) =7 7(63.7) ≥9 3 (27.3)	12.9mo
Ryan Phillips^5^	2020	U.S.A	Prospective, Phase III Trial	ORIOLE	NA	Observation	18	68 (64–76)	7 (3–17)	= 6 0 =7 8 (44) = 8 1 (6) = 9 8 (44) =10 1(16)	SABR	36	68 (61–70)	6 (2–13)	= 6 3 (8) =7 22(61) = 8 4 (11) = 9 7 (19) =10 0	18.8 (IQR, 5.8–35.0) mo
Piet Ost^6^	2018	Belgium	Prospective, Phase II Trial	NCT01558427	NA	Surveillance	31	63.3 (47–79)	12.1 (2.5–36.2)	≤6 10 (32.3) =7 11 (32.3) ≥8 10 (32.3)	metastasis- directed therapy (MDT)	31	60.8 (43–75)	22.0 (3.5–114.0)	≤6 4 (12.9) =7 17 (54.8) ≥8 10 (32.3)	36 (IQR, 27.6–45.6) mo
Giulio Francolini^7^	2023	Italy	Prospective, Phase III Trial	ARTO- NCT03449719	NA	Abiraterone acetate and prednisone	23	69 (65–76.2)	2.55 (IQR 1.2–6.11)	NA	Abiraterone acetate and prednisone+SBRT	19	73 (IQR 66–78)	2. 91 (IQR 1.6–6.6)	NA	6mo
Andrew J Armstrong^8^	2023	U.S.A	Prospective, Phase III Trial	ARCHES- NCT02677896	NA	PBO + ADT	221	70 (64–75)	2.4 (0.4–11.0)	<8 78 (35)	ENZA + ADT	244	70 (65–75)	3.2 (0.6–15.5)	<8 81 (33)	44.6mo
Luca Boeri^9^	2021	U.S.A	Single-center	NA	NA	ADT	121	NA	7.5 (5.1–11.7)	6 10 (13.5) 7 42 (56.7) ≥8 22 (29.8)	EBRT (external beam RT)	178	NA	6.5 (4.9–10.2)	6 3 (4.7) 7 35 (55.5) ≥8 25 (39.8)	46.7(IQR, 32.4–61.3) mo
Berna Akkus Yildirim^10^	2019	Turkey	Retrospective	NE	NE	Abiraterone	62	74 (50–92)	60.8 ± 48.9	≤7 25(51) >7 37 (65)	Abiraterone+ curative radiotherapy	44	73 (47–92)	51.4 ± 48.4	≤7 24(49) >7 20 (35)	14.2 (IQR, 2.3–54.9) mo
Karim Fizazi^11^	2022	U.S.A	Prospective, Phase III Trial	PEACE-1	T1 23 T2 109 T3 356 T4 133	SOC	296	66 (59–72)	11 (3–55)	≤7 133 (23%) 8–10 441 (77%)	SOC plus abiraterone	292	67 (61–72)	14 (3–62)	≤7 145 (25%) 8–10 429 (75%)	3.5 years (IQR 2.8–4.6)
Matthew P Deek^12^	2022	U.S.A	Prospective	STOMP ORIOLE	T1 6 T2 45 T3 61 T4 4	Observation	49	NA	5.93 (1.1–10.1)	=6 5 (10.2) =7 22 (44.9) =8 4 (8.2) =9 16 (32.7) =10 2 (4.1)	MDT (RT)	67	70.40 (62.60–73.80)	5.0 (1.9–11.1)	=6 6 (9) =7 38 (56.7) =8 9 (13.4) =9 14 (20.9)	52.5 mo (range, 5.8–92.0 mo).
Liselotte M S Boevé^13^	2019	Netherlands	Prospective	Horrad	NA	ADT	216	67 (61–71)	NA	=6 7 (3) =7 64 (30) =8 65 (30) =9 72 (33) =10 7 (3)	ADT+EBRT	216	67 (62–71).	NA	=6 7 (3) =7 66 (31) = 8 48 (22) =9 85 (39) =10 9 (4)	47 (IQR, 36–68) mo

**Notes.**

ADT androgen-deprivation therapy ENZA enzalutamide EBRT external beam radiation therapy GS Gleason score IQR interquartile range NA not available PSA prostate-specific antigen PSMAgRT PSMA-PET/CT-guided intensification of radiation therapy RLT radical local therapy RT radiation therapy SABR stereotactic ablative radiotherapy SOC Standard of Care

1 Tang et al. (2023).

2 Dai et al. (2022).

3 Sooriakumaran et al. (2022).

4 Petit et al. (2023).

5 Phillips et al. (2020).

6 Ost et al. (2018).

7 Francolini et al. (2023).

8 Armstrong et al. (2023).

9 Boeri et al. (2021).

10 Yildirim et al. (2019).

11 Fizazi et al. (2022).

12 Deek et al. (2022).

13 Boevé et al. (2019).

**Figure 2:**
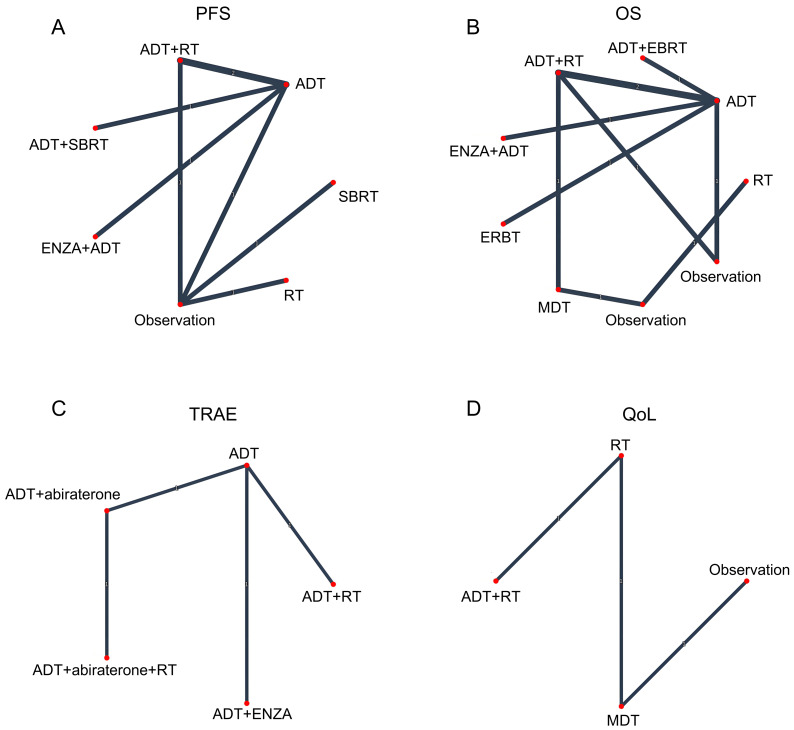
Network geometry of treatment comparisons for (A) progression-free survival (PFS), (B) overall survival (OS), (C) grade ≥3 treatment-related adverse events (TRAEs), and (D) quality of life (QoL) outcomes.

The risk of bias for each trial is reported in Fig. S3. For network meta-analysis purposes, the ADT arm from each trial served as the common comparator across all treatment comparisons (Fig. 2).

### Progression-free survival

**Figure 3:**
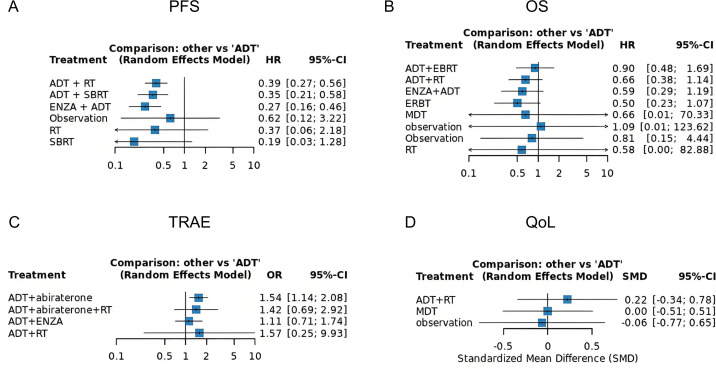
Forest plots demonstrating comparative treatment effects on (A) PFS, (B) OS, (C) grade ≥3 TRAEs, and (D) QoL across included studies.

Eight randomized trials evaluating the impact of various treatment modalities on progression free survival (PFS) were included in this NMA, with the treatment network illustrated in Fig. 2A. The funnel plot demonstrated a roughly symmetrical distribution of included studies, indicating no substantial publication bias (Fig. S1A). Compared to ADT alone, significant reductions in disease progression risk were observed with combination therapies: ADT+ RT (HR = 0.39, 95% CI [0.27–0.56]), ADT + SBRT (HR = 0.35, 95% CI [0.21–0.58]), and ENZA + ADT (HR = 0.27, 95% CI [0.16–0.46]). In contrast, RT alone failed to demonstrate significant clinical benefit (HR = 0.60, 95% CI [0.31–1.13]) (Fig. 3A). Interestingly, while SUCRA analysis initially suggested ADT monotherapy as the most likely optimal treatment, comprehensive league table evaluation revealed that ADT combined with RT may provide superior PFS outcomes. Furthermore, ADT + SBRT also showed considerable potential for improving PFS (Table 2, Fig. S2A).

**Table 2:** League table with pooled standardized mean difference for PFS, OS, TREA, QoL.

**HR with 95% confidence interval on PFS**							
**ADT**	2.59 (1.80, 3.74)	2.86 (1.73, 4.71)	3.70 (2.18, 6.28)	1.00 (0.10, 10.00)	.	.	
**2.56 (1.78, 3.69)**	**ADT + RT**	.	.	1.00 (0.10, 10.00)	.	.	
**2.86 (1.73, 4.71)**	1.12 (0.60, 2.07)	**ADT + SBRT**	.	.	.	.	
**3.70 (2.18, 6.28)**	1.45 (0.76, 2.75)	1.30 (0.63, 2.68)	**ENZA + ADT**	.	.	.	
1.60 (0.31, 8.24)	0.62 (0.12, 3.22)	0.56 (0.10, 3.11)	0.43 (0.08, 2.42)	**Observation**	1.67 (0.87, 3.18)	3.33 (1.23, 9.05)	
2.67 (0.46, 15.52)	1.04 (0.18, 6.06)	0.93 (0.15, 5.82)	0.72 (0.11, 4.53)	1.67 (0.87, 3.18)	**RT**	.	
**HR with 95% confidence interval on OS**							
**ADT**	1.11 (0.59, 2.08)	1.54 (0.88, 2.71)	1.69 (0.84, 3.40)	2.00 (0.94, 4.27)	.	.	1.00 (0.09, 10.74)
1.11 (0.59, 2.08)	**ADT+EBRT**	.	.	.	.	.	.
1.52 (0.87, 2.66)	1.37 (0.59, 3.17)	**ADT+RT**	.	.	1.00 (0.01, 103.70)	.	1.00 (0.09, 10.74)
1.69 (0.84, 3.40)	1.53 (0.60, 3.90)	1.11 (0.46, 2.71)	**ENZA+ADT**	.	.	.	.
2.00 (0.94, 4.27)	1.80 (0.67, 4.82)	1.31 (0.51, 3.36)	1.18 (0.42, 3.31)	**ERBT**	.	.	.
1.52 (0.01, 163.36)	1.37 (0.01, 153.34)	1.00 (0.01, 103.70)	0.90 (0.01, 101.50)	0.76 (0.01, 86.83)	**MDT**	0.60 (0.30, 1.22)	.
0.91 (0.01, 103.37)	0.82 (0.01, 96.98)	0.60 (0.01, 65.64)	0.54 (0.00, 64.19)	0.46 (0.00, 54.91)	0.60 (0.30, 1.22)	**observation**	.
1.23 (0.23, 6.77)	1.11 (0.18, 6.82)	0.81 (0.15, 4.44)	0.73 (0.12, 4.58)	0.62 (0.10, 3.98)	0.81 (0.01, 113.63)	1.35 (0.01, 199.15)	**Observation**
**OR with 95% confidence interval on TRAEs**							
**ADT**	0.65 (0.48, 0.88)	.	0.90 (0.58, 1.40)	0.63 (0.10, 4.00)			
**0.65 (0.48, 0.88)**	**ADT+abiraterone**	1.08 (0.56, 2.09)	.	.			
0.70 (0.34, 1.45)	1.08 (0.56, 2.09)	**ADT+abiraterone+RT**	.	.			
0.90 (0.58, 1.40)	1.39 (0.81, 2.37)	1.28 (0.55, 2.98)	**ADT+ENZA**	.			
0.63 (0.10, 4.00)	0.98 (0.15, 6.32)	0.90 (0.12, 6.51)	0.71 (0.11, 4.69)	**ADT+RT**			
**SMD with 95% confidence interval on QoL**							
**ADT**	−0.22 [−0.78; 0.34]	0.00 [−0.51; 0.51]	.				
**−0.22 [−0.78; 0.34]**	**ADT+RT**	.	.				
**−0.00 [−0.51; 0.51]**	0.22 [−0.53; 0.98]	**MDT**	0.06 [−0.43; 0.56]				
**0.06 [−0.65; 0.77]**	0.28 [−0.62; 1.19]	0.06 [−0.43; 0.56]	**observation**				

### Overall survival

Our analysis incorporated seven studies reporting HR with confidence intervals, supplemented by two virtual studies (Observation, HR = 1.0, 95% CI = 0.1–10) to ensure network connectivity. The resulting network diagram demonstrated nine treatment modalities forming direct or indirect connections through common comparators (ADT or Observation) (Fig. 2B). Funnel plot analysis revealed a symmetrical distribution of studies, indicating no substantial publication bias, with the inclusion of virtual studies not affecting outcome interpretation (Fig. S1B). Forest plot and league table analyses of treatment comparisons showed that none of the alternative therapies significantly improved OS compared to ADT, although these differences did not reach statistical significance (Fig. 3B, Table 2). Consequently, ADT emerged with the highest SUCRA ranking, suggesting it may represent the optimal treatment choice for OS outcomes (Fig. S2B).

### Treatment-related adverse events

Our safety evaluation incorporated four studies examining grade ≥3 TRAEs, with the network diagram demonstrating all treatment modalities interconnected through ADT, forming a single coherent network (Fig. 2C). Funnel plot analysis revealed no significant publication bias (Fig. S1C). Forest plot results indicated that ADT + abiraterone significantly increased TRAE risk compared to ADT monotherapy (OR = 1.54, 95% CI [1.14–2.08]), while ADT + RT showed a non-significant trend (OR = 1.57, 95% CI [0.25–9.30]), with the wide confidence interval likely reflecting limited sample size (Fig. 3C). SUCRA analysis and league table rankings established the following safety hierarchy: ADT (most favorable) >ADT+abiraterone >ADT+ENZA >ADT+abiraterone+RT >ADT+RT, confirming ADT monotherapy’s superior safety profile, though its therapeutic limitations must be carefully considered (Fig. S2C, Table 2).

### Quality of Life

The network meta-analysis of QoL outcomes incorporated two studies evaluating four treatment approaches (ADT+RT, ADT, MDT, Observation). Network geometry and funnel plot analysis demonstrated all treatments formed a connected network through virtual comparisons, with no evidence of publication bias (Fig. 2D, Fig. S1D). Forest plot results indicated considerable uncertainty in determining QoL differences among treatment modalities (Fig. 3D). SUCRA analysis and league table rankings revealed ADT+RT achieved the highest probability of being optimal (SUCRA 74.3%), suggesting its potential as the most favorable QoL improvement strategy, while Observation ranked lowest as anticipated (Fig. S2D, Table 2). These findings suggest ADT+RT may offer modest QoL benefits, though the limited evidence precludes definitive conclusions due to the small number of included studies and inherent methodological constraints of the indirect comparisons.

### Risk of bias

The risk of bias assessment for all included randomized controlled trials is shown in Fig. S3. Overall, 13 trials were considered to have a low overall risk of bias. Four trials were considered to be at high risk of bias in outcome assessment. Almost all randomized controlled trials were considered to be at low risk of bias at D1, D3, D4, and D6.

## Discussion

Oligometastatic prostate cancer (omPCa) represents an intermediate stage of cancer dissemination between localized and widely metastatic disease. With dramatic improvements in staging—particularly through advanced functional imaging such as PSMA-PET and robotic-assisted (RA) targeted biopsies (RA-TB)—omPCa is now more frequently detected, both at primary diagnosis and in oligorecurrent disease (Patel et al., 2020). Singh et al. (2004) investigated the correlation between survival and the number of metastatic lesions observed in each prostate cancer patient. They found that men with ≤5 lesions had survival rates comparable to those without metastases, and these rates were significantly higher than those of patients with >5 lesions. These findings underscore the growing rationale for aggressive, metastasis-directed therapy (MDT) in omPCa. Retrospective and prospective studies suggest that surgical interventions (cytoreductive prostatectomy (CRP), salvage lymph node dissection (SLND), or metastasectomy) can achieve favorable short-term oncologic outcomes and local disease control (von Deimling et al., 2022). However, high-quality evidence on long-term oncological benefits remains limited. For de novo oligometastatic hormone-sensitive prostate cancer (omHSPC), ongoing trials are evaluating the role of primary tumor resection, while in metachronous disease, SLND should be reserved for carefully selected patients within a multimodal approach to eradicate detectable disease and prolong progression-free survival (PFS) (von Deimling et al., 2022). Notably, clinicians should remain vigilant for the potential presence of neuroendocrine prostate cancer (NEPC) in metastatic prostate cancer (mPCa) cases. This rare but highly aggressive variant necessitates immediate therapeutic strategy modifications (Wishahi, 2024).

Recent advances in cancer genomics, sequencing technologies, and functional genomics have led to the development of novel therapeutic approaches, including immunotherapies that have demonstrated promising efficacy (Gillessen et al., 2023; Ashrafizadeh et al., 2022). However, further research is required to fully elucidate their clinical benefits (Miyahira et al., 2022). For oligometastatic prostate cancer (omPCa), current management strategies typically involve local consolidation therapy (*e.g.*, stereotactic body radiotherapy (SBRT)) targeting the primary tumor, followed by metastatic lesion treatment and subsequent systemic hormonal therapy. Clinical evidence from completed trials (NCT02192788, NCT02680587, NCT02264379) available on ClinicalTrials.gov has demonstrated sustained clinical benefits of SBRT in oligometastatic castration-sensitive prostate cancer (omCSPC). Ongoing investigations through currently recruiting studies (NCT04610372 [PROMPT], NCT04115007, NCT04599686) are expected to provide additional insights. While enhanced recovery after surgery (ERAS) protocols have been successfully implemented across various surgical specialties (Wishahi, Kamal & Hedaya, 2024), surgical intervention is generally contraindicated in metastatic prostate cancer. Emerging evidence suggests that primary tumor treatment may reduce the need for palliative interventions in locally advanced cases (Heidenreich, Pfister & Porres, 2015; Won et al., 2013) and potentially delay the initiation of systemic therapies such as androgen deprivation therapy (ADT)—an approach that could significantly improve quality of life (Taylor, Canfield & Du, 2009). This consideration is particularly relevant given that while ADT remains a cornerstone of metastatic prostate cancer treatment, it is associated with substantial adverse effects and may, in some patient populations, negatively impact overall survival (Taylor, Canfield & Du, 2009; Sammon et al., 2015). Our study findings align with these observations, demonstrating that ADT—whether administered alone or in combination with SBRT—showed the least significant improvement in PFS among treated patients.

Retrospective studies indicate that interventions like radical prostatectomy and targeted radiotherapy for either local or metastatic lesions can be performed in metastatic settings with minimal risk of severe toxic effects. Heidenreich, Pfister & Porres (2015) reported no Clavien grade IV or V complications in men undergoing radical prostatectomy, and the incidence of grade I–III complications was similar to or better than controls. Additionally, palliative intervention was needed in 28.9% (11 out of 38) of control patients compared to none who underwent radical prostatectomy. Moreover, 21 (91.3%) patients reported postoperative continence, requiring zero or one pad per day. A retrospective multi-institutional analysis of radical prostatectomy in distant metastasis settings showed similarly promising results (Sooriakumaran et al., 2016). The rates of complications, readmission, and reoperation were 20.8%, 3.8%, and 1.9%, respectively, compared to 19.4%, 3.0%, and 2.3%, respectively, in open cases for standard indications (Tewari et al., 2012). These treatments appear to reduce the requirement for subsequent palliative care, though there is insufficient data to reliably conclude their impact on survival. Therefore, a standardized clinical protocol for managing patients with metastatic prostate cancer would be a valuable clinical tool.

Our network meta-analysis (NMA) of 13 trials (*n* = 2, 524) underscores the superiority of ADT combined with RT (ADT + RT) in both progression-free survival (PFS: SUCRA = 51.4%, HR = 0.39) and QoL (OS: SUCRA = 74.3%, HR = 0.22), aligning with landmark trials like ENZAMET and ARCHES (Sweeney et al., 2023; Armstrong et al., 2019). However, combination therapies, including ADT + abiraterone, increased treatment-related adverse events (TRAEs) by 54% (OR = 1.54), while ranked lowest in safety (SUCRA = 25.8%). These findings highlight a critical dilemma—aggressive regimens maximize survival but exacerbate toxicity, whereas ADT monotherapy, though safer (SUCRA = 79.8%), offers limited efficacy.

Our study has limitations reflective of the broader field. First, the heterogeneous definition of “oligometastatic” disease complicates cross-trial comparisons; standardized criteria (such as ≤5 lesions on PSMA-PET) are urgently needed. Second, approximated hazard ratios for studies reporting median survival times (such as Yildirim et al. (2019)) introduce bias, emphasizing the need for raw Kaplan–Meier curves in publications. Third, QoL outcomes were inconclusive due to sparse data (ADT + RT: SMD = 0.22, 95% CI [−0.34–0.78]), underscoring the imperative to integrate patient-reported outcomes (PROs) in future trials. Finally, the combination of ADT with abiraterone demonstrated significantly elevated adverse event risk, necessitating rigorous clinical monitoring. Interestingly, our analysis revealed the counterintuitive finding that ADT+abiraterone+RT showed better safety outcomes than ADT+RT alone. We hypothesize this paradoxical result may stem from abiraterone’s potential to enhance RT feasibility through two mechanisms: (1) significant tumor downstaging prior to radiation, and (2) reduced tumor volume facilitating post-treatment recovery. The apparent TRAE reduction likely reflects decreased tumor progression rather than true treatment-related toxicity mitigation. Despite these limitations, the results of our analysis provide strong evidence for the treatment of oligometastatic prostate cancer and can serve as an important reference.

## Conclusion

This network meta-analysis of 13 studies (2,524 patients) found that ADT + RT and ADT + SBRT significantly improved PFS (HR = 0.39 and 0.35 *vs.* ADT alone) but showed no OS benefit. ADT monotherapy had the lowest toxicity, while ADT + abiraterone increased grade ≥3 TRAEs (OR = 1.54). Limited data suggested ADT + RT may improve QoL.

##  Supplemental Information

10.7717/peerj.19819/supp-1 Supplemental Information 1 PRISMA checklist

10.7717/peerj.19819/supp-2 Supplemental Information 2 Risk of bias assessment for included randomized controlled trials

10.7717/peerj.19819/supp-3 Supplemental Information 3 Funnel plots assessing publication bias for (A) PFS, (B) OS, (C) grade ≥ 3 TRAEs, and (D) QoL outcomes

10.7717/peerj.19819/supp-4 Supplemental Information 4 SUCRA (surface under the cumulative ranking curve) plots presenting treatment rankings for (A) PFS, (B) OS, (C) grade ≥ 3 TRAEs, and (D) QoL

10.7717/peerj.19819/supp-5 Supplemental Information 5 Statement of how disagreements were resolved, and identify the referee

10.7717/peerj.19819/supp-6 Supplemental Information 6 Rationale for conducting the NMA

## Additional Information and Declarations

### Data Availability

The following information was supplied regarding data availability: This is a systematic review/meta-analysis.

## References

[ref-1] Armstrong AJ, Iguchi T, Azad AA, Villers A, Alekseev B, Petrylak DP, Szmulewitz RZ, Alcaraz A, Shore ND, Holzbeierlein J, Gomez-Veiga F, Rosbrook B, Zohren F, Haas GP, Gourgiotti G, El-Chaar N, Stenzl A (2023). The efficacy of enzalutamide plus androgen deprivation therapy in oligometastatic hormone-sensitive prostate cancer: a *post hoc* analysis of ARCHES. European Urology.

[ref-2] Armstrong AJ, Szmulewitz RZ, Petrylak DP, Holzbeierlein J, Villers A, Azad A, Alcaraz A, Alekseev B, Iguchi T, Shore ND, Rosbrook B, Sugg J, Baron B, Chen L, Stenzl A (2019). ARCHES: a randomized, phase III study of androgen deprivation therapy with enzalutamide or placebo in men with metastatic hormone-sensitive prostate cancer. Journal of Clinical Oncology.

[ref-3] Ashrafizadeh M, Paskeh MDA, Mirzaei S, Gholami MH, Zarrabi A, Hashemi F, Hushmandi K, Hashemi M, Nabavi N, Crea F, Ren J, Klionsky DJ, Kumar AP, Wang Y (2022). Targeting autophagy in prostate cancer: preclinical and clinical evidence for therapeutic response. Journal of Experimental & Clinical Cancer Research.

[ref-4] Boeri L, Sharma V, Kwon E, Stish BJ, Davis BJ, Karnes RJ (2021). Oligorecurrent prostate cancer treated with metastases-directed therapy or standard of care: a single-center experience. Prostate Cancer and Prostatic Diseases.

[ref-5] Boevé LMS, Hulshof MCCM, Vis AN, Zwinderman AH, Twisk JWR, Witjes WPJ, Delaere KPJ, Moorselaar RJAV, Verhagen PCMS, van Andel G (2019). Effect on survival of androgen deprivation therapy alone compared to androgen deprivation therapy combined with concurrent radiation therapy to the prostate in patients with primary bone metastatic prostate cancer in a prospective randomised clinical trial: data from the HORRAD trial. European Urology.

[ref-6] Cornford P, Bellmunt J, Bolla M, Briers E, De Santis M, Gross T, Henry AM, Joniau S, Lam TB, Mason MD, Van der Poel HG, Van der Kwast TH, Rouvière O, Wiegel T, Mottet N, EAU-ESTRO-SIOG Guidelines on Prostate Cancer (2017). Part II: treatment of relapsing, metastatic, and castration-resistant prostate cancer. European Urology.

[ref-7] Dai B, Zhang S, Wan FN, Wang HK, Zhang JY, Wang QF, Kong YY, Ma XJ, Mo M, Zhu Y, Qin XJ, Lin GW, Ye DW (2022). Combination of androgen deprivation therapy with radical local therapy *versus* androgen deprivation therapy alone for newly diagnosed oligometastatic prostate cancer: a phase II randomized controlled trial. European Urology Oncology.

[ref-8] Deek MP, Van der Eecken K, Sutera P, Deek RA, Fonteyne V, Mendes AA, Decaestecker K, Kiess AP, Lumen N, Phillips R, De Bruycker A, Mishra M, Rana Z, Molitoris J, Lambert B, Delrue L, Wang H, Lowe K, Verbeke S, Van Dorpe J, Bultijnck R, Villeirs G, De Man K, Ameye F, Song DY, DeWeese T, Paller CJ, Feng FY, Wyatt A, Pienta KJ, Diehn M, Bentzen SM, Joniau S, Vanhaverbeke F, De Meerleer G, Antonarakis ES, Lotan TL, Berlin A, Siva S, Ost P, Tran PT (2022). Long-term outcomes and genetic predictors of response to metastasis-directed therapy *versus* observation in oligometastatic prostate cancer: analysis of STOMP and ORIOLE trials. Journal of Clinical Oncology.

[ref-9] Fizazi K, Foulon S, Carles J, Roubaud G, McDermott R, Fléchon A, Tombal B, Supiot S, Berthold D, Ronchin P, Kacso G, Gravis G, Calabro F, Berdah JF, Hasbini A, Silva M, Thiery-Vuillemin A, Latorzeff I, Mourey L, Laguerre B, Abadie-Lacourtoisie S, Martin E, El Kouri C, Escande A, Rosello A, Magne N, Schlurmann F, Priou F, Chand-Fouche ME, Freixa SV, Jamaluddin M, Rieger I, Bossi A (2022). Abiraterone plus prednisone added to androgen deprivation therapy and docetaxel in *de novo* metastatic castration sensitive prostate cancer (PEACE-1): a multicentre, open-label, randomised, phase 3 study with a 2 × 2 factorial design. Lancet.

[ref-10] Francolini G, Allegra AG, Detti B, Di Cataldo V, Caini S, Bruni A, Ingrosso G, D’Angelillo RM, Alitto AR, Augugliaro M, Triggiani L, Parisi S, Facchini G, Banini M, Simontacchi G, Desideri I, Meattini I, Valicenti RK, Livi L, ARTO Working Group members (2023). Stereotactic body radiation therapy and abiraterone acetate for patients affected by oligometastatic castrate resistant prostate cancer: a randomized phase II trial (ARTO). Journal of Clinical Oncology.

[ref-11] Gillessen S, Bossi A, Davis ID, De Bono J, Fizazi K, James ND, Mottet N, Shore N, Small E, Smith M, Sweeney C, Tombal B, Antonarakis ES, Aparicio AM, Armstrong AJ, Attard G, Beer TM, Beltran H, Bjartell A, Blanchard P, Briganti A, Bristow RG, Bulbul M, Caffo O, Castellano D, Castro E, Cheng HH, Chi KN, Chowdhury S, Clarke CS, Clarke N, Daugaard G, De Santis M, Duran I, Eeles R, Efstathiou E, Efstathiou J, Ngozi Ekeke O, Evans CP, Fanti S, Feng FY, Fonteyne V, Fossati N, Frydenberg M, George D, Gleave M, Gravis G, Halabi S, Heinrich D, Herrmann K, Higano C, Hofman MS, Horvath LG, Hussain M, Jereczek-Fossa BA, Jones R, Kanesvaran R, Kellokumpu-Lehtinen PL, Khauli RB, Klotz L, Kramer G, Leibowitz R, Logothetis CJ, Mahal BA, Maluf F, Mateo J, Matheson D, Mehra N, Merseburger A, Morgans AK, Morris MJ, Mrabti H, Mukherji D, Murphy DG, Murthy V, Nguyen PL, Oh WK, Ost P, O’Sullivan JM, Padhani AR, Pezaro C, Poon DMC, Pritchard CC, Rabah DM, Rathkopf D, Reiter RE, Rubin MA, Ryan CJ, Saad F, Pablo Sade J, Sartor OA, Scher HI, Sharifi N, Skoneczna I, Soule H, Spratt DE, Srinivas S, Sternberg CN, Steuber T, Suzuki H, Sydes MR, Taplin ME, Tilki D, Türkeri L, Turco F, Uemura H, Uemura H, Ürün Y, Vale CL, van Oort I, Vapiwala N, Walz J, Yamoah K, Ye D, Yu EY, Zapatero A, Zilli T, Omlin A (2023). Management of patients with advanced prostate cancer, part I: intermediate-/high-risk and locally advanced disease, biochemical relapse, and side effects of hormonal treatment: report of the advanced prostate cancer consensus conference 2022. European Urology.

[ref-12] Heidenreich A, Pfister D, Porres D (2015). Cytoreductive radical prostatectomy in patients with prostate cancer and low volume skeletal metastases: results of a feasibility and case-control study. Journal D Urologie.

[ref-13] Higgins JP, Altman DG, Gøtzsche PC, Jüni P, Moher D, Oxman AD, Savovic J, Schulz KF, Weeks L, Sterne JA (2011). The Cochrane Collaboration’s tool for assessing risk of bias in randomised trials. Bmj.

[ref-14] Miyahira AK, Zarif JC, Coombs CC, Flavell RR, Russo JW, Zaidi S, Zhao D, Zhao SG, Pienta KJ, Soule HR (2022). Prostate cancer research in the 21st century; report from the 2021 Coffey-Holden prostate cancer academy meeting. Prostate.

[ref-15] Ost P, Reynders D, Decaestecker K, Fonteyne V, Lumen N, De Bruycker A, Lambert B, Delrue L, Bultijnck R, Claeys T, Goetghebeur E, Villeirs G, De Man K, Ameye F, Billiet I, Joniau S, Vanhaverbeke F, De Meerleer G (2018). Surveillance or metastasis-directed therapy for oligometastatic prostate cancer recurrence: a prospective, randomized, multicenter phase II trial. Journal of Clinical Oncology.

[ref-16] Page MJ, McKenzie JE, Bossuyt PM, Boutron I, Hoffmann TC, Mulrow CD, Shamseer L, Tetzlaff JM, Akl EA, Brennan SE, Chou R, Glanville J, Grimshaw JM, Hróbjartsson A, Lalu MM, Li T, Loder EW, Mayo-Wilson E, McDonald S, McGuinness LA, Stewart LA, Thomas J, Tricco AC, Welch VA, Whiting P, Moher D (2021). The PRISMA 2020 statement: an updated guideline for reporting systematic reviews. Systematic Reviews.

[ref-17] Patel MI, Muter S, Vladica P, Gillatt D (2020). Robotic-assisted magnetic resonance imaging ultrasound fusion results in higher significant cancer detection compared to cognitive prostate targeting in biopsy naive men. Translational Andrology and Urology.

[ref-18] Petit C, Delouya G, Taussky D, Barkati M, Lambert C, Beauchemin MC, Clavel S, Mok G, Paré AG, Nguyen TV, Duplan D, Keu KV, Saad F, Juneau D, Ménard C (2023). PSMA-PET/CT-guided intensification of radiation therapy for prostate cancer (PSMAgRT): findings of detection rate, effect on cancer management, and early toxicity from a phase 2 randomized controlled trial. International Journal of Radiation Oncology, Biology, Physics.

[ref-19] Phillips R, Shi WY, Deek M, Radwan N, Lim SJ, Antonarakis ES, Rowe SP, Ross AE, Gorin MA, Deville C, Greco SC, Wang H, Denmeade SR, Paller CJ, Dipasquale S, DeWeese TL, Song DY, Wang H, Carducci MA, Pienta KJ, Pomper MG, Dicker AP, Eisenberger MA, Alizadeh AA, Diehn M, Tran PT (2020). Outcomes of observation *vs* stereotactic ablative radiation for oligometastatic prostate cancer: the ORIOLE phase 2 randomized clinical trial. JAMA Oncology.

[ref-20] Sammon JD, Abdollah F, Reznor G, Pucheril D, Choueiri TK, Hu JC, Kim SP, Schmid M, Sood A, Sun M, Kibel AS, Nguyen PL, Menon M, Trinh QD (2015). Patterns of declining use and the adverse effect of primary androgen deprivation on all-cause mortality in elderly men with prostate cancer. European Urology.

[ref-21] Singh D, Yi WS, Brasacchio RA, Muhs AG, Smudzin T, Williams JP, Messing E, Okunieff P (2004). Is there a favorable subset of patients with prostate cancer who develop oligometastases?. International Journal of Radiation Oncology, Biology, Physics.

[ref-22] Sooriakumaran P, Karnes J, Stief C, Copsey B, Montorsi F, Hammerer P, Beyer B, Moschini M, Gratzke C, Steuber T, Suardi N, Briganti A, Manka L, Nyberg T, Dutton SJ, Wiklund P, Graefen M (2016). A multi-institutional analysis of perioperative outcomes in 106 men who underwent radical prostatectomy for distant metastatic prostate cancer at presentation. European Urology.

[ref-23] Sooriakumaran P, Wilson C, Rombach I, Hassanali N, Aning J, Lamb AD, Cathcart P, Eden C, Ahmad I, Rajan P, Sridhar A, Bryant RJ, Elhage O, Cook J, Leung H, Soomro N, Kelly J, Nathan S, Donovan JL, Hamdy FC (2022). Feasibility and safety of radical prostatectomy for oligo-metastatic prostate cancer: the testing radical prostatectomy in men with prostate cancer and oligo-Metastases to the bone (TRoMbone) trial. BJU International.

[ref-24] Sweeney CJ, Martin AJ, Stockler MR, Begbie S, Cheung L, Chi KN, Chowdhury S, Frydenberg M, Horvath LG, Joshua AM, Lawrence NJ, Marx G, McCaffrey J, McDermott R, McJannett M, North SA, Parnis F, Parulekar W, Pook DW, Reaume MN, Sandhu SK, Tan A, Tan TH, Thomson A, Vera-Badillo F, Williams SG, Winter D, Yip S, Zhang AY, Zielinski RR, Davis ID (2023). Testosterone suppression plus enzalutamide *versus* testosterone suppression plus standard antiandrogen therapy for metastatic hormone-sensitive prostate cancer (ENZAMET): an international, open-label, randomised, phase 3 trial. The Lancet Oncology.

[ref-25] Tang C, Sherry AD, Haymaker C, Bathala T, Liu S, Fellman B, Cohen L, Aparicio A, Zurita AJ, Reuben A, Marmonti E, Chun SG, Reddy JP, Ghia A, McGuire S, Efstathiou E, Wang J, Wang J, Pilie P, Kovitz C, Du W, Simiele SJ, Kumar R, Borghero Y, Shi Z, Chapin B, Gomez D, Wistuba I, Corn PG (2023). Addition of metastasis-directed therapy to intermittent hormone therapy for oligometastatic prostate cancer: the EXTEND phase 2 randomized clinical trial. JAMA Oncology.

[ref-26] Taylor LG, Canfield SE, Du XL (2009). Review of major adverse effects of androgen-deprivation therapy in men with prostate cancer. Cancer.

[ref-27] Tewari A, Sooriakumaran P, Bloch DA, Seshadri-Kreaden U, Hebert AE, Wiklund P (2012). Positive surgical margin and perioperative complication rates of primary surgical treatments for prostate cancer: a systematic review and meta-analysis comparing retropubic, laparoscopic, and robotic prostatectomy. European Urology.

[ref-28] Tosoian JJ, Gorin MA, Ross AE, Pienta KJ, Tran PT, Schaeffer EM (2017). Oligometastatic prostate cancer: definitions, clinical outcomes, and treatment considerations. Nature Reviews Urology.

[ref-29] Von Deimling M, Rajwa P, Tilki D, Heidenreich A, Pallauf M, Bianchi A, Yanagisawa T, Kawada T, Karakiewicz PI, Gontero P, Pradere B, Ploussard G, Rink M, Shariat SF (2022). The current role of precision surgery in oligometastatic prostate cancer. ESMO Open.

[ref-30] Wishahi M (2024). Treatment-induced neuroendocrine prostate cancer and de novo neuroendocrine prostate cancer: identification, prognosis and survival, genetic and epigenetic factors. World Journal of Clinical Cases.

[ref-31] Wishahi M, Kamal NM, Hedaya MS (2024). Enhanced recovery after surgery: progress in adapted pathways for implementation in standard and emerging surgical settings. World Journal of Clinical Cases.

[ref-32] Won AC, Gurney H, Marx G, De Souza P, Patel MI (2013). Primary treatment of the prostate improves local palliation in men who ultimately develop castrate-resistant prostate cancer. BJU International.

[ref-33] Yildirim BA, Onal C, Kose F, Oymak E, Sedef AM, Besen AA, Aksoy S, Guler OC, Sumbul AT, Muallaoglu S, Mertsoylu H, Ozyigit G (2019). Outcome of loco-regional radiotherapy in metastatic castration-resistant prostate cancer patients treated with abiraterone acetate. Strahlentherapie und Onkologie.

[ref-34] Ying WW, Ding ZS, Zhang T, Wang JF, Chen X, Zhou XF, Qong YQ (2022). Radiotherapy and endocrine therapy for oligometastatic prostate cancer efficacy: a systematic review and network meta-analysis. Inplasy Protocol.

