# Supplementary figures and images for: Surgery, radiotherapy and endocrine therapy for oligometastatic prostate cancer efficacy: a systematic review and network meta-analysis
Source: PeerJ. 2025 Aug 29;13:e19819. doi: 10.7717/peerj.19819 (PMC12401018; doi:10.7717/peerj.19819)

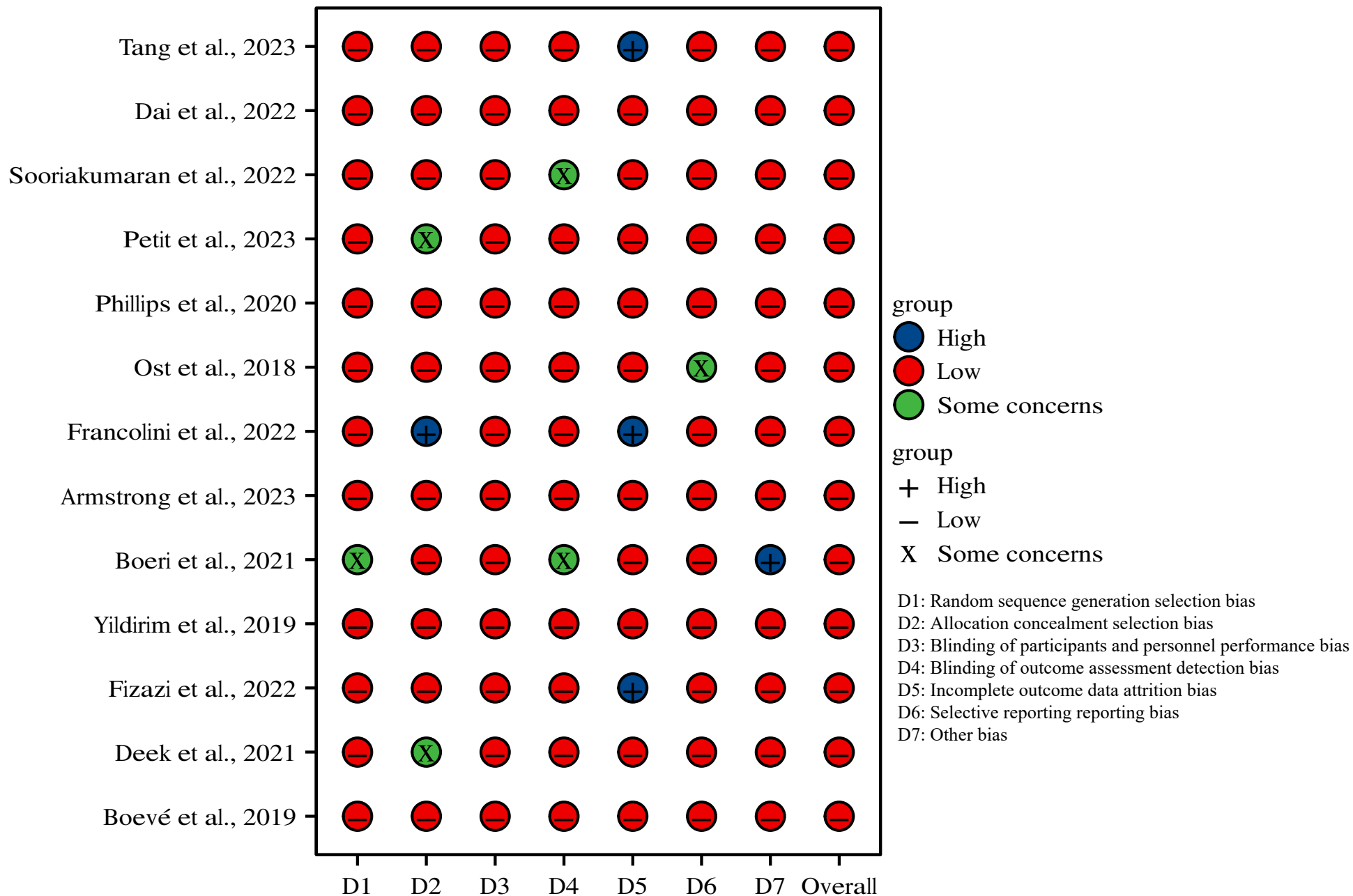

Supplement: Supplemental Information 2 [file peerj-13-19819-s002.pdf]

A

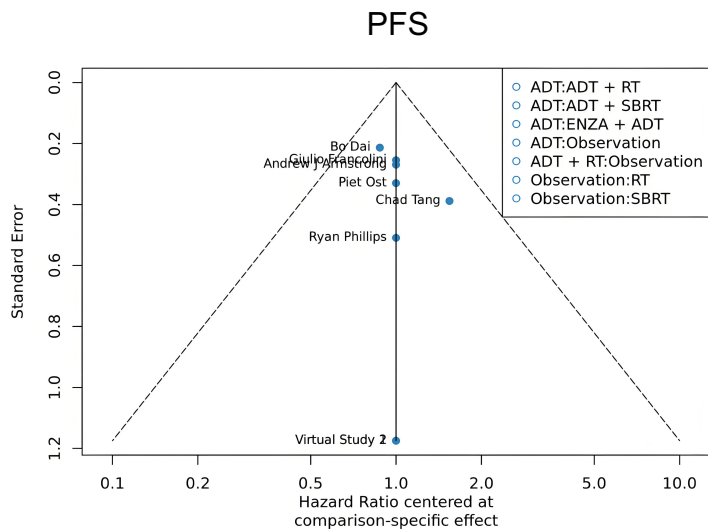

B

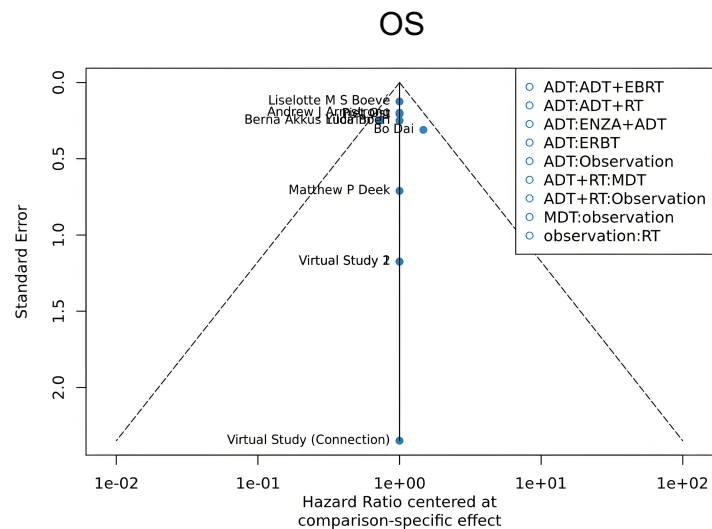

C

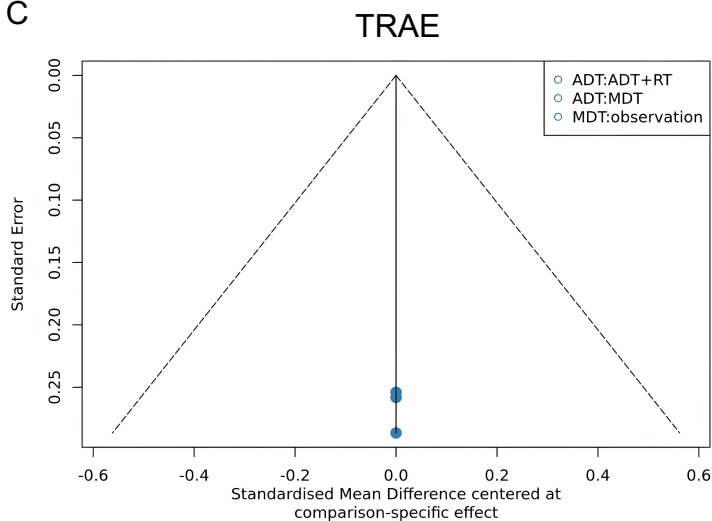

D

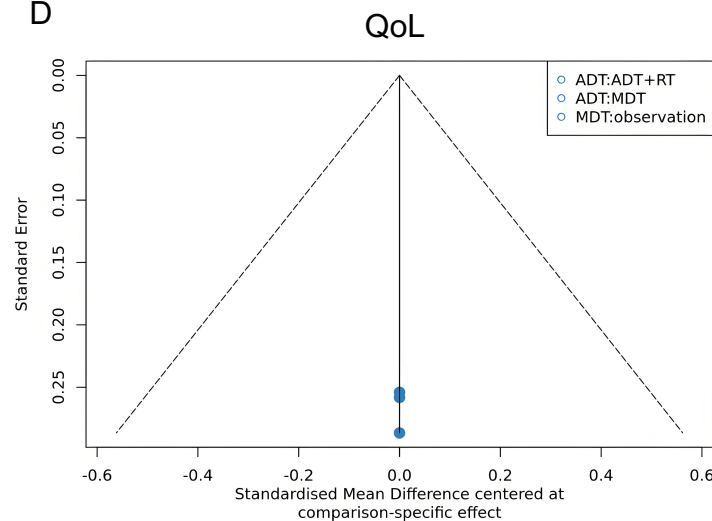

Supplement: Supplemental Information 3 [file peerj-13-19819-s003.pdf]

A

PFS

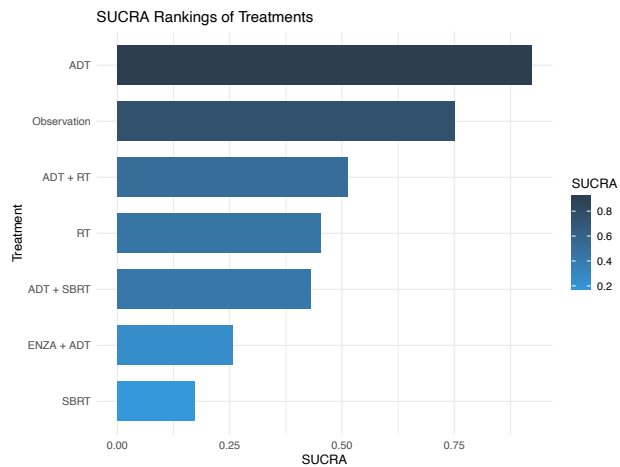

B

OS

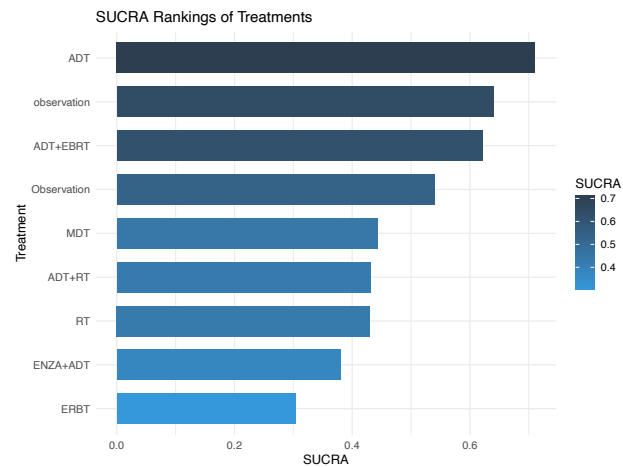

C

TRAE

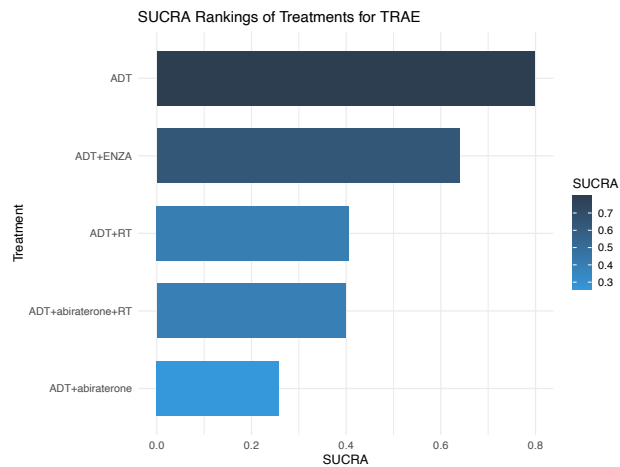

D

QoL

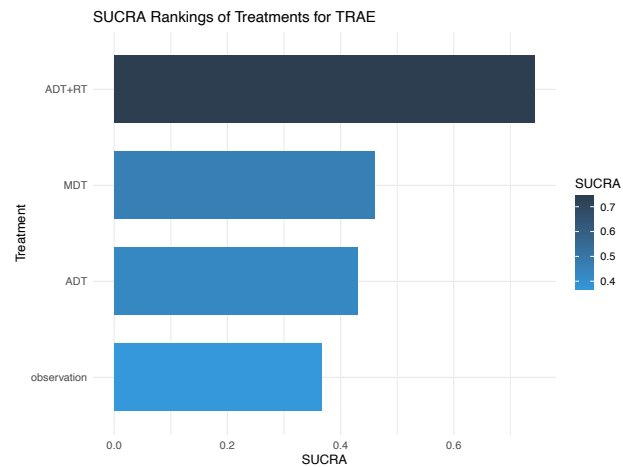

Supplement: Supplemental Information 4 [file peerj-13-19819-s004.pdf]
