# Supplementary material for: Surgery, radiotherapy and endocrine therapy for oligometastatic prostate cancer efficacy: a systematic review and network meta-analysis
Source: PeerJ. 2025 Aug 29;13:e19819. doi: 10.7717/peerj.19819 (PMC12401018; doi:10.7717/peerj.19819)
Supplement: Supplemental Information 5 [file peerj-13-19819-s005.docx]

**Background:** The management of metastatic prostate cancer has shifted toward early combination therapies rather than androgen-deprivation therapy (ADT) alone, yet the comparative efficacy and safety of these treatments remain uncertain. While randomized controlled trials (RCTs) have explored various interventions for oligometastatic prostate cancer (omPCa), definitive conclusions regarding their prognostic benefits and patient-centered outcomes are lacking.

**Objective:** This network meta-analysis (NMA) aims to systematically evaluate the benefits and risks of different treatment strategies for omPCa, focusing on overall survival (OS), progression-free survival (PFS), treatment-related adverse events (TRAEs), and quality of life (QoL).

**Methods:** We conducted a systematic search of Medline, EMBASE, and Cochrane databases for RCTs published up to October 1, 2024. Individual patient data from 13 eligible trials (n=2,524) were analyzed using network meta-analysis. The study was registered with PROSPERO (CRD42022370203).

**Primary Outcomes:** The primary outcomes assessed were OS, PFS, TRAEs (grade ≥3), and QoL. Treatment effects were quantified using hazard ratios (HRs) for survival outcomes and odds ratios (ORs) for toxicity, with surface under the cumulative ranking curve (SUCRA) scores used to rank interventions.

**Results:** ADT combined with radiotherapy (ADT+RT, HR=0.39, 95% CI 0.27–0.56) or stereotactic body radiotherapy (ADT+SBRT, HR=0.35, 95% CI 0.21–0.58) significantly improved PFS compared to ADT alone, though no OS benefit was observed. ADT monotherapy had the lowest toxicity risk, while ADT+abiraterone increased adverse events (OR=1.54). Limited QoL data suggested potential improvement with ADT+RT (SUCRA 74.3%). Most trials demonstrated low bias risk, though heterogeneity and small sample sizes for some comparisons necessitate cautious interpretation.

**Conclusion:** These findings support ADT+RT/SBRT for PFS improvement in omPCa but underscore the absence of OS benefits and the trade-offs in toxicity. Further research is needed to optimize survival outcomes and treatment tolerability, particularly with emerging therapies not yet evaluated in RCTs.

**Registration:** This study is registered with PROSPERO, CRD42022370203.
