# Supplementary material for: Surgery, radiotherapy and endocrine therapy for oligometastatic prostate cancer efficacy: a systematic review and network meta-analysis
Source: PeerJ. 2025 Aug 29;13:e19819. doi: 10.7717/peerj.19819 (PMC12401018; doi:10.7717/peerj.19819)
Supplement: Supplemental Information 6 [file peerj-13-19819-s006.docx]

**RATIONALE for Conducting the Systematic Review / Meta-Analysis**

**Why It Is Necessary to Conduct This Systematic Review and Network Meta-Analysis**

In recent years, the treatment approach for metastatic prostate cancer has evolved significantly, with early combination therapies increasingly being favored over androgen-deprivation therapy (ADT) alone. Despite the availability of various treatment options, their relative effectiveness remains uncertain. Randomized controlled trials (RCTs) have explored a range of treatments for oligometastatic prostate cancer, but clear conclusions regarding their prognostic benefits have not been established. Therefore, this study aims to quantify the benefits of different treatment approaches through a systematic review and network meta-analysis (NMA), providing a more reliable basis for clinical decision-making.

**Key Points of Necessity**

**1.Uncertainty in Treatment Strategies:**

Current treatment options for metastatic prostate cancer, particularly oligometastatic prostate cancer, are still debated. While early combination therapies have shown promise, the specific combinations and their long-term outcomes are not well-documented.

A systematic review and NMA can integrate results from multiple RCTs, offering a comprehensive evaluation of the relative effectiveness of different treatment strategies.

**2.Support for Clinical Decision-Making:**

This study will assess the impact of various treatments on overall survival (OS), progression-free survival (PFS), treatment-related adverse events (TRAEs), and quality of life (QoL). These outcomes are crucial for clinicians in selecting the most appropriate treatment for their patients.

For patients with oligometastatic prostate cancer, determining the most effective treatment approach can significantly improve their prognosis and quality of life.

**3.Multi-Dimensional Efficacy Evaluation:**

Traditional meta-analyses are limited to comparing only two treatment options. In contrast, NMA can simultaneously compare multiple treatments, providing a more holistic view of treatment efficacy.

By analyzing individual patient data from 13 eligible trials involving 2,524 patients, this study will offer more precise insights into the specific effects of different treatment combinations, thus supporting the development of personalized treatment plans.

**4.Updating and Supplementing Existing Knowledge:**

This NMA will cover the latest trial data up to October 1, 2024, from Medline, EMBASE, and Cochrane databases, ensuring the timeliness and reliability of the results.

Previous meta-analyses and systematic reviews may have missed some relevant RCTs. This study aims to fill these gaps and provide a more comprehensive evidence base.

**5.Promoting Improvement in Clinical Practice:**

By clearly elucidating the effectiveness and safety of various treatment options, this study will help clinicians better understand the characteristics of different treatments, enabling more informed clinical decisions.

The findings of this study will also contribute to the development of clinical guidelines and standards, promoting the standardization and personalization of metastatic prostate cancer treatment.

In summary, this systematic review and network meta-analysis is necessary to provide a comprehensive, multi-dimensional evaluation of the efficacy and safety of different treatment approaches for metastatic prostate cancer. The results will offer reliable evidence to support clinical decision-making and improve patient outcomes and quality of life.
